# Predicting Response Trajectories during Cognitive-Behavioural Therapy for Panic Disorder: No Association with the *BDNF* Gene or Childhood Maltreatment

**DOI:** 10.1371/journal.pone.0158224

**Published:** 2016-06-29

**Authors:** Martí Santacana, Bárbara Arias, Marina Mitjans, Albert Bonillo, María Montoro, Sílvia Rosado, Roser Guillamat, Vicenç Vallès, Víctor Pérez, Carlos G. Forero, Miquel A. Fullana

**Affiliations:** 1 Department of Mental Health, Consorci Sanitari de Terrassa, Terrassa, Spain; 2 Department of Psychiatry and Legal Medicine, Universitat Autònoma de Barcelona, Cerdanyola del Vallès, Spain; 3 Anthropology Unit, Department of Animal Biology, Universitat de Barcelona, Barcelona, Spain; 4 CIBERSAM (Centro de Investigaciones Biomédicas en Red de Salud Mental), Instituto de Salud Carlos III, Madrid, Spain; 5 Clinical Neuroscience, Max Planck Institute of Experimental Medicine, Göttingen, Germany; 6 Department of Psychobiology and Methodology of Health Sciences, Universitat Autònoma de Barcelona, Cerdanyola del Vallès, Spain; 7 Institute of Neuropsychiatry and Addictions, Hospital del Mar, Barcelona, Spain; 8 CIBERESP (Centro de Investigaciones Biomédicas en Red, Epidemiología y Salud Pública), Instituto de Salud Carlos III, Madrid, Spain; 9 Health Services Research Group, IMIM (Institut Hospital del Mar d'Investigacions Mèdiques), Barcelona, Spain; 10 Department of Experimental and Life Sciences (DCEXS), Universitat Pompeu Fabra, Barcelona, Spain; West China Second Hospital, Sichuan University, CHINA

## Abstract

**Background:**

Anxiety disorders are highly prevalent and result in low quality of life and a high social and economic cost. The efficacy of cognitive-behavioural therapy (CBT) for anxiety disorders is well established, but a substantial proportion of patients do not respond to this treatment. Understanding which genetic and environmental factors are responsible for this differential response to treatment is a key step towards “personalized medicine”. Based on previous research, our objective was to test whether the *BDNF* Val66Met polymorphism and/or childhood maltreatment are associated with response trajectories during exposure-based CBT for panic disorder (PD).

**Method:**

We used Growth Mixture Modeling to identify latent classes of change (response trajectories) in patients with PD (N = 97) who underwent group manualized exposure-based CBT. We conducted logistic regression to investigate the effect on these trajectories of the *BDNF* Val66Met polymorphism and two different types of childhood maltreatment, abuse and neglect.

**Results:**

We identified two response trajectories (“high response” and “low response”), and found that they were not significantly associated with either the genetic (*BDNF* Val66Met polymorphism) or childhood trauma-related variables of interest, nor with an interaction between these variables.

**Conclusions:**

We found no evidence to support an effect of the *BDNF* gene or childhood trauma-related variables on CBT outcome in PD. Future studies in this field may benefit from looking at other genotypes or using different (e.g. whole-genome) approaches.

## Introduction

The high prevalence of the anxiety disorders is a major public health concern. Twelve-month prevalence rates have been estimated at 18.1% in the US [1] and 13.6% in Europe [2]. In addition, anxiety disorders have a marked impact on the quality of life of those affected [3], and cause a huge social and economic burden. At the European level, Olesen *et al*. [4] quantified the total annual cost of anxiety disorders as 74.4 billion euros.

The efficacy of cognitive-behavioural therapy (CBT) for anxiety disorders is well established from randomised controlled trials [5], and is recommended by current clinical guidelines [6, 7]. However, a substantial proportion of patients do not respond to this treatment [8], and identifying the factors underlying this differential treatment response is now a research priority. In this context, genetic and environmental predictors of outcome have gained attention [9, 10] and will make a key contribution towards “personalized medicine” [11]. However, it remains to be tested whether results of basic research can be translated into clinical practice.

The prediction of treatment response based on genetic background has a long history in pharmacotherapy, but is a relatively new development in the field of psychological treatment. The term “therapygenetics” was coined a few years ago to indicate the prediction of psychological treatment outcomes from genetic markers [10, 12, 13]. Most research in this area has focused on testing the effects of specific single nucleotide polymorphisms (SNP) on response to CBT in anxiety and depressive disorders. Although the field is in its infancy, initial evidence indicates that specific SNPs may improve the prediction of CBT outcomes (i.e. a decrease in measures of severity). The most widely assessed SNP have been the serotonin transporter gene functional length polymorphism (5HTTLPR), and the functional Val66Met (rs6265) polymorphism in the brain-derived neurotrophic factor (*BDNF*) gene [9, 10].

Both preclinical and clinical data suggest that Val66Met is involved in response to CBT (and more specifically, to exposure-based interventions) for anxiety and related disorders. Val66Met, which involves substitution of valine (Val) with methionine (Met), affects intracellular trafficking and secretion of *BDNF*, and Met carriers show decreased activity-dependent secretion of this neurotrophin [14]. Consistent with animal studies, this variation may be associated with anxiety or fear in humans [15]. Recent research suggests that extinction learning, which is the experimental model for exposure therapy, may be modulated by genetic variation in *BDNF*, and both mouse and human carriers of the Met variant show impaired extinction learning [16]. Moreover, humans carriers show a different pattern of activation of brain regions that are essential for fear extinction (including the ventromedial prefrontal cortex and the amygdala), which is consistent with impaired extinction learning [16, 17]. Preliminary translation of these basic results is provided by two recent studies showing that among adults with post-traumatic stress disorder (PTSD) [18] and obsessive-compulsive disorder (OCD) [19], those with the Met allele had a poorer response to exposure-based CBT than those with the Val/Val genotype. However, this association was not replicated in a study in adults with social anxiety disorder (SAD) who received internet-based or group CBT [20], or in a study in children with mixed anxiety disorders who were receiving CBT [21].

Regarding environmental factors, childhood maltreatment has been the focus of extensive research. Beyond its established role as a risk factor for many mental disorders [22, 23], childhood maltreatment may also influence treatment outcomes. There is a well established association between childhood maltreatment and poor response to treatment for depression, and this association seems to be independent of treatment modality (pharmacological or psychological) [24]. However, this issue has not been investigated in detail for anxiety disorders, and very few studies have considered whether specific forms of maltreatment are relevant. To our knowledge, only two studies in patients with SAD have examined the impact of specific types of childhood maltreatment on CBT outcomes in anxiety disorders [25, 26]. Alden *et al*. [25] found that parental abuse increased risk of poor treatment outcome, while Bruce *et al*. [26] found no association between specific types of childhood maltreatment and response to CBT.

There has been at least one preliminary attempt to combine genetic and clinical variables to predict outcomes of psychological treatment in children with anxiety disorders [9]. To our knowledge, however, the role of genetics and early developmental experiences such as maltreatment in predicting the outcome of CBT in adults has not yet been explored.

Our aim was to study whether the *BDNF* Val66Met polymorphism and/or childhood maltreatment were associated with the outcome of exposure-based CBT for panic disorder (PD) in a naturalistic setting. Based on growth mixture models (GMM), we operationalized treatment outcome with trajectories of change in panic symptoms. Trajectory analyses have recently been used in psychological treatment research to identify patterns of change and to examine treatment effects in different patients subgroups [27–32]. This approach has several methodological advantages and is probably more clinically informative [28, 30, 33, 34].

Based on previous research, we predicted the existence of distinct response trajectories in patients receiving exposure-based CBT, and we hypothetised that genetic variation (presence of the *BDNF* Met allele) and childhood maltreatment (both abuse and neglect) would be associated with a less favourable response trajectory.

## Method

### Participants

**Fig 1:**
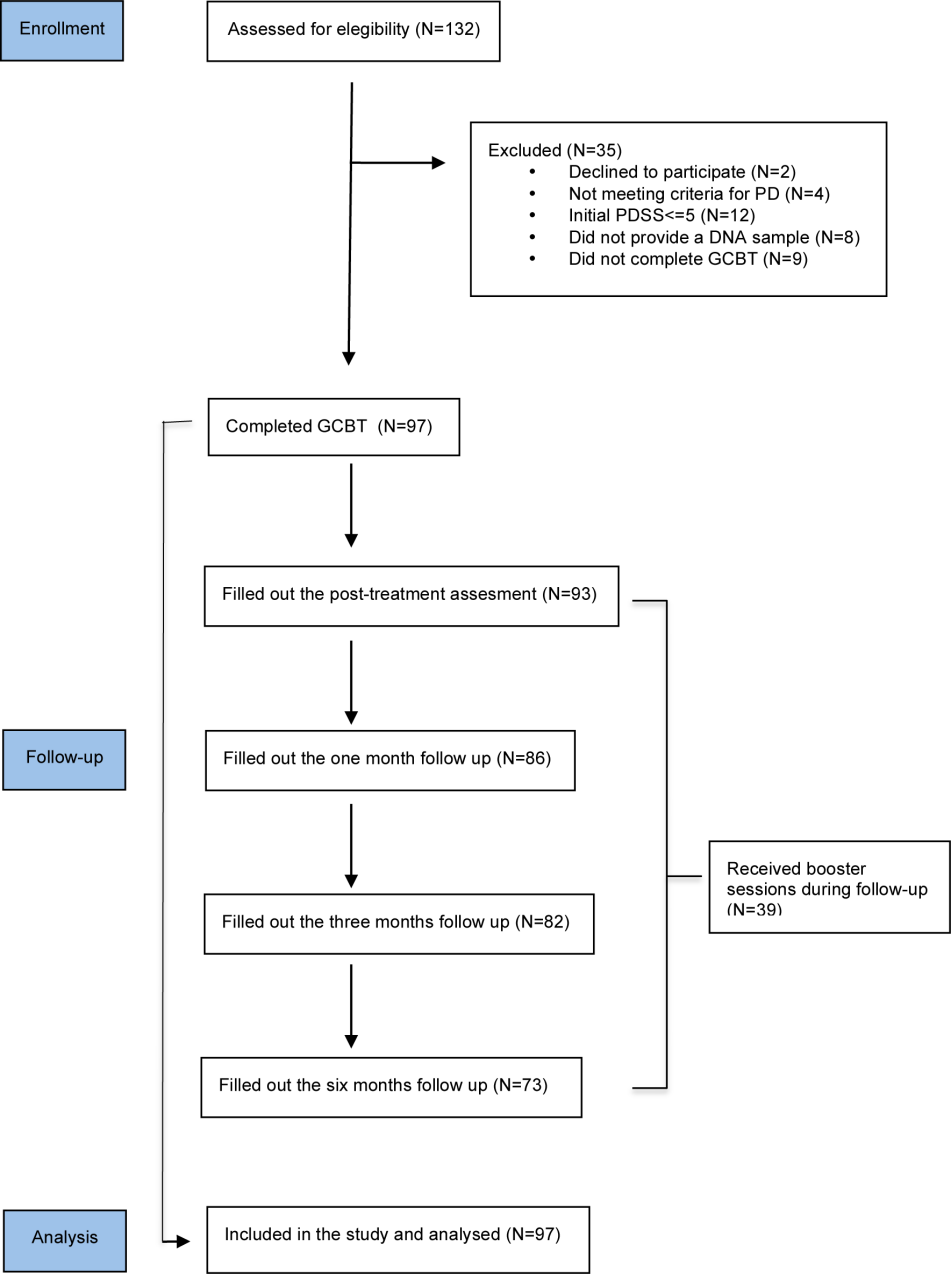
Consort flow diagram.

Our sample consisted of 97 patients who completed (i.e., attended ≥ 8 sessions) a 9-week intervention of manualized exposure-based group CBT (GCBT) for PD at one of two treatment centres in Barcelona (Spain) between February 2011 and January 2014. During the recruitment period, 132 patients were assessed, and the following exclusion criteria were applied: age <18 or >60 years; presence or history of any organic mental disorder, bipolar disorder or psychosis; substance abuse or dependence in the previous three months (except for nicotine); intellectual disability; and language barriers. A Consort flow diagram of the recruitment process is shown in Fig 1.

All participants had a primary diagnosis of PD (89.7% with agoraphobia) according to the DSM-IV-TR [35], and had a score of ≥6 on the Spanish self-report version [36] of the *Panic Disorder Severity Scale* (PDSS-SR) [37]. Diagnosis was established by one experienced clinician using a semi-structured interview, and was confirmed by another clinician using the validated Spanish version [38] of the *Mini International Neuropsychiatric Interview* (MINI) [39].

The final sample included 61 women (62.9%), and the mean age was 36.19 years (range 18–60, *SD* = 9.23). Twenty-two participants (22.7%) presented one or more additional axis-I disorders. The most common comorbidities were SAD (7.2%), specific phobia (6.2%), generalized anxiety disorder (3.1%), and others (6.2%). On admission, eighty-two patients (84.5%) were taking psychiatric medication (mainly antidepressants) continually for at least two months prior to beginning treatment, and 40 (41.2%) were taking PRN benzodiazepines. Participants were asked to maintain their medication regime during the GCBT. Table 1 shows a demographic and clinical summary of the sample.

**Table 1:** Demographic and clinical characteristics of the sample (N = 97).

		% / Mean (*sd*)
Gender		
	Men	37.1%
	Women	62.9%
Age (years)		36.19 (9.23)
Marital status		
	Single	30.5%
	Married/in a relationship	69.5%
Education level		
	Elementary school	28.4%
	High school	45.3%
	University	26.3%
Unemployed		45.8%
Age of onset (panic symptoms)		25.58 (9.13)
Duration of current episode (in years)		4.13 (5.58)
Presence of agoraphobia		89.7%
Axis-I comorbidity		22.7%
Pharmacological treatment		
	No medication	15.5%
	Antidepressant + benzodiazepine	41.2%
	Antidepressant	19.6%
	Benzodiazepine	23.7%
*BDNF* Val66Met genotypes		
	Val-Val	61.9%
	Met carriers	38.1%
CTQ-SF scores		
	Emotional abuse	9.64 (5.24)
	Physical abuse	6.44 (3.30)
	Sexual abuse	5.69 (1.56)
	Emotional neglect	10.66 (4.82)
	Physical neglect	6.59 (2.46)

M = mean, *sd* = standard deviation, CTQ-SF = Childhood Trauma Questionnaire-Short Form

The study fulfilled the principles of the World Medical Association Declaration of Helsinki and was approved by the Clinical Research Ethical Committee of the Parc de Salut Mar (Barcelona, Spain) and by the Clinical Research Ethical Committee of the Consorci Sanitari de Terrassa (Barcelona, Spain). All participants provided voluntary informed written consent.

### Assessment

The MINI (Spanish version 5.0.0) is a brief structured interview that evaluates the presence of Axis I disorders according to DSM-IV criteria [39], and is a widely used instrument in clinical and research settings. The Spanish version of the MINI has shown to have sound psychometric properties [40].

The self-report version of the *Panic Disorder Severity Scale* (PDSS-SR) is a 7-item instrument that assesses the frequency of panic attacks, associated distress, anticipatory anxiety, agoraphobic fear and avoidance, body-sensation fear and avoidance, and impaired work and social function [37] during the previous week. Each item is scored on a 5-point ordinal scale (0–4), and the total score ranges between 0 and 28. The Spanish PDSS-SR has shown excellent internal consistency, good test-retest reliability, adequate convergent and divergent validity, and good sensitivity to change [36].

The *Childhood Trauma Questionnaire-Short Form* (CTQ-SF) is a 28-item instrument that measures five types of childhood maltreatment: emotional abuse, physical abuse, sexual abuse, emotional neglect, and physical neglect [41, 42]. Each item ranges from 1 to 5, and the total score of each subscale ranges from 5 to 25. Following previous research [43], childhood maltreatment was grouped into two main types: childhood abuse (including emotional, physical and sexual abuse) and childhood neglect (including emotional and physical neglect). Childhood abuse and childhood neglect were calculated by summing the items of the corresponding subscales. The Spanish CTQ-SF has been shown to be a reliable and valid instrument [44].

### Treatment

Group CBT consisted of nine one-hour sessions administered on a weekly basis. Groups were conducted by a main therapist (a PhD level clinical psychologist) and a co-therapist (a clinical psychology trainee or psychiatric nurse), and ranged from 4 to 7 patients each. Treatment was mannualized and the protocol was a modified version of the *Panic Control Treatment* by Barlow and Craske [45], adapted by the last author. Sessions 1 to 3 were devoted to education about anxiety and panic; sessions 4 and 5 consisted of in-session interoceptive exposure exercises, and progressive withdrawal of safety behaviors; and sessions 6 to 9 focused on graded exposure to avoided situations and activities. The treatment included homework exercises that varied depending on the phase of therapy, and did not include specific cognitive therapy tasks. To maximize treatment integrity and adherence to the protocol, fortnightly meetings between the main therapists were scheduled and checklists with the items to be covered at each session were provided to the co-therapists. Thirty-nine patients (40.2%) had at least one individual “booster” session during the six month follow-up period. Previous research has established the effectiveness of this treatment protocol [46].

### Procedure

At the beginning of each session and at one, three and six months follow-up, participants completed the PDSS-SR to assess panic symptoms during the previous week. Additionally, participants also completed the CTQ-SF during the initial assessment session.

### Genetic analyses

Genomic DNA was extracted from buccal mucosa on a cotton swab, or from blood samples using the Real Extraction DNA Kit (Durviz S.L.U., Valencia, Spain). The rs6265 SNP (Val66Met) of the *BDNF* gene was determined using the Taqman 5‘ exonuclease assay -Applied Biosystems (AB)- and genotyped using AB TaqMan technology. The probe for genotyping the rs6265 was ordered through the TaqMan SNP Genotyping assays AB assay-on-demand service (code C_11592758_10). The final volume of the polymerase chain reaction (PCR) reaction was 5 mL, which contained 10 ng of genomic DNA, 2.5 ml of TaqMan Master Mix, and 0.125 ml of 40x genotyping assay. The cycling parameters were as follows: 95°C for 10 min, followed by 40 cycles of denaturation at 92°C for 15 s and annealing/extension at 60°C for 1 min. PCR plates were read on an ABI PRISM 7900HT instrument with SDS v2.1 software (AB). The genotype frequencies of rs6265-*BDNF* were in Hardy-Weinberg equilibrium (χ^2^ = 0.53; *p* = 0.467).

### Statistical analyses

In the first step, we used growth mixture models (GMM) analysis to empirically identify latent subpopulations of patients with similar change trajectories (‘trajectory classes’) in panic symptoms, as measured using the PDSS-SR (baseline, plus 9 weekly ratings, plus three follow-up sessions). In GMM, trajectory classes are characterized by two parameters: intercept (i.e. initial severity) and slope (i.e. rate of change). GMM allows us to test for an *a priori* unknown number of latent subpopulations with different intercepts and slopes, as well as class-specific variations around these parameters.

To determine the optimal number of trajectory classes, we compared different linear models, starting with a one-class model (i.e. assuming that all participants followed the same trajectory) and then adding classes in each subsequent run. For each model, allocation of individuals was based on maximum posterior probability [47]. Models were estimated with 2000 random starting values, using MPLUS software (version 7.0). To decide on the number of trajectory classes, we examined the following indices of goodness of fit: Akaike Information Criterion (AIC), Bayesian Information Criterion (BIC), sample-size adjusted BIC (SSBIC) and the bootstrap likelihood ratio test (BLRT). The model with the lowest SSBIC was chosen [48]. The BLRT was used to assess differences in log-likelihood between models with k and k+1 classes, where p<0.05 indicates the benefit of adding an additional class [49]. We also computed the entropy value for each class solution. In addition to the indices of goodness of fit, the optimal number of latent trajectory classes was also based on group membership posterior probabilities, distinctiveness of classes and how well the PDSS-SR profiles of subjects from one class matched the class average. For all intercept and slope coefficients, we report unstandardized coefficients.

In the second step of the analyses, we conducted logistic regression analysis to test whether genetic background (presence/absence of the *BDNF* 66Met allele) and trauma-related variables (childhood abuse and childhood neglect) predicted class membership. To control for the potential effect of pharmacological treatment, this variable was included in the model as a covariate. Finally, the model also included variables for interaction effects between childhood abuse and *BDNF* genotype, and between childhood neglect and *BDNF* gentoype.

## Results

### Genetic and trauma-related variables

Carriers of one or two *BDNF*66Met alleles were combined as a ‘‘Met-carrier group”, consistent with previous studies [18, 19, 50]. The genotype frequencies for the *BDNF* Val66Met polymorphism were: 31.8% (N = 37) Met allele carriers (including 6 Met/Met homozygotes), and 61.9% (N = 60) Val-Val homozygotes. In line with previous reports [51–53], we evaluated the prevalence of childhood maltreatment was evaluated using the following cut-off scores: emotional abuse ≥9, physical abuse ≥8, sexual abuse ≥6, emotional neglect ≥10 and physical neglect ≥8. In the current sample, 53 participants (55.2%) had been exposed to at least one type (emotional, physical, or sexual) of childhood abuse, and 51 (53.7%) to at least one type (emotional or physical) of childhood neglect.

### Identification of trajectory classes

Preliminary sensitivity analyses showed that the models including and excluding participants with more than 3 missing data points gave the same class structures. Since missing data did not affect results substantially, two participants with missing data were excluded, leaving 95 participants for the the trajectory analyses.

Table 2 shows the fit indices for models with 1 to 3 trajectory classes for the PDSS-SR. BLRT was only significant in the change between the 1-class and the 2-class models, which was not significantly different to the the 3-class model. The 2-class solution was therefore selected because it had the lowest SSBIC and a high entropy, which was 0.85, over the recommended cut-off of 0.80 [54].

**Table 2:** Model fit indices, *p*-value in Bootstrap Likelihood Ratio Test and entropy for up to three latent classes.

Model	AIC	BIC	SSBIC	BLRT difference (number of parameters)	*p*	Entropy
1-class	6549.88	6629.05	6531.18	--	--	
2-class	6547.61	6652.32	6522.80	23.46 (4)	0.037	0.851
3-class	6583.56	6636.18	6548.89	16.85 (4)	0.092	0.873

AIC = Akaike Information Criterion, BIC = Bayesian Information Criterion, SSBIC = sample-size adjusted BIC, BLRT = Bootstrap Likelihood Ratio Test

**Fig 2:**
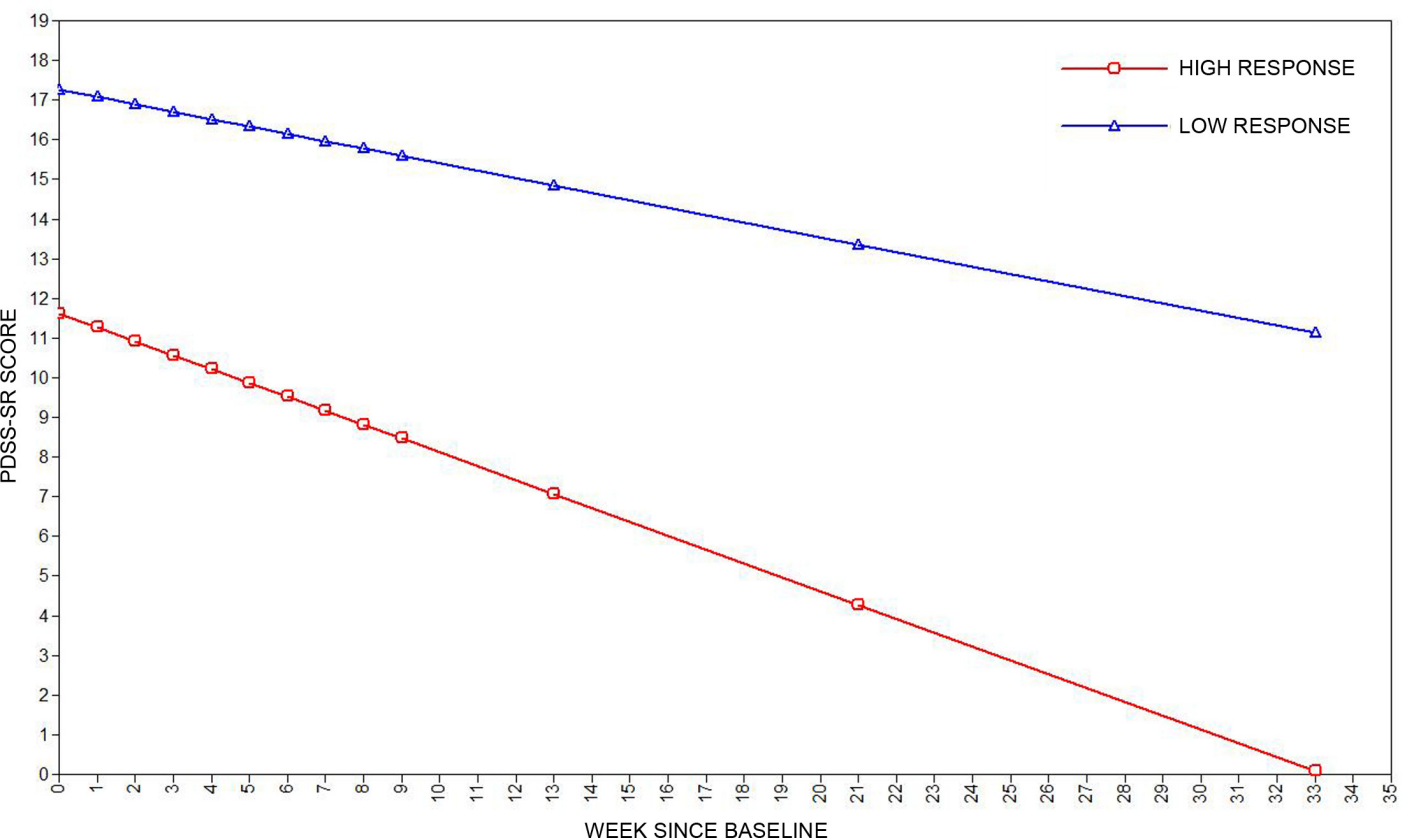
Estimated mean scores for panic symptoms (PDSS-SR) in the two-class model during exposure-based CBT and follow-up.

Fig 2 shows that one class (“high response”) included patients (N = 76; 80.3% of the sample) with lower baseline panic symptoms (intercept = 12.12, SE = 2.061, *p*<0.01) and a tendency to improve over time (slope = -0.329, SE = 0.12, *p* = 0.01). The other class (“low response”) included patients (N = 19; 19.7% of the sample) with greater panic symptoms at baseline (intercept = 18.36, SE = 2.76, *p*<0.01) and which did not improve across time (slope = -0.17, SE = 0.15, *p* = 0.26).

### Effects of genetic background and trauma on trajectory classes

Table 3 shows that neither the *BDNF* Val66Met polymorphism nor the specific types of trauma analysed (abuse and neglect) predicted membership of any specific trajectory class. We found no significant evidence to suggest that trajectory class membsership was affected by gene-environment interaction between the *BDNF* Met allele and childhood abuse, nor between the *BDNF* Met allele and childhood neglect.

**Table 3:** Main effects and interactions of genetic and trauma-related variables on trajectory class membership.

	B	SE	*p*
***Main effects***			
*BDNF* Met allele	0.078	0.550	0.887
Childhood abuse	0.003	0.046	0.946
Childhood neglect	-0.058	0.058	0.319
***Interaction effects***			
BDNF*childhood abuse	-0.060	0.098	0.544
BDNF*childhood neglect	0.059	0.123	0.631

SE = standard error

## Discussion

To our knowledge, this is the first study to evaluate the effects of the *BDNF* Val66Met polymorphism and childhood maltreatment as possible predictors of CBT outcome in PD (assessed using trajectories of change). We found no evidence that either *BDNF* Val66Met or childhood maltreatment were significant predictors of CBT outcome.

In our sample PD patients followed two different trajectories during treatment. Many patients (80.3%) showed a tendency to improve as a result of CBT, whereas a significant proportion (19.7%) did not. While there is a general paucity of data on trajectories of change during CBT for PD, our results are consistent with data from a previous study that highlighted a group of PD patients with high baseline symptoms that tend not to improve with CBT [28]. We observed a similar percentage of patients with this non-remitting course (19.7%) to that reported by Lutz et al. (17.2%).

We did not find a significant association between the *BDNF* Val66Met polymorphism and CBT treatment outcome. In light of previous reports, our results show that there is no compelling evidence to suggest that this genetic variation can be used to predict response to exposure-based CBT. In previous studies in adults, PTSD [18] and OCD patients [19] who carried the Met allele showed a poorer response than Val/Val homozygotes, although effects of *BDNF* in the OCD study were apparent in the percentage of responders, but not in the pre- to post-treatment severity scores. In addition, no association has been found between the *BDNF* Met allele and CBT outcome in SAD [20] or in mixed child anxiety disorders [21]. Thus, based on ours and previous studies, it seems that the relatively well established association between the *BDNF* gene and learning and memory, and specifically with fear extinction [16, 55], does not translate easily into clinical practice. One possible explanation is that the BNDF effect may be observed in very controlled experiments, but may be diluted among the multiple variables involved in clinical studies [56]. In an extensive review of the association between the *BDNF* Val66Met polymorphism and mental disorders, Notaras and van den Buuse [57] highlight some of the possible methodological shortcomings as an explanation for previous inconsistent results, including small sample sizes and ethnicity-specific effects. It may also be the case that the *BNDF* gene provokes a poorer response in some anxiety-related disorders but not in others. For example, Hedman *et al*. [12] have recently proposed that exposure might have a more decisive role in some anxiety-related disorders (e.g., PTSD) than in others (e.g., SAD).

Another possibility is that other polymorphisms (rather than the one studied here) may be more important for predicting CBT outcome. A recent large study (N = 829) did not replicate the previously reported association [12] between the functional serotonin transporter promoter polymorphism and CBT outcome [58]. The authors of this study recommended abandoning the single-variant approach and moving towards a whole-genome array-based therapygenomics approach. A recent study from the same group also found no evidence of an effect of hypothalamic-pituitary-adrenal axis-related genes on response to CBT [59]. These authors also invesitgated epigenetic factors (changes in DNA methylation) and suggested that epigenetic variables may be a better predictor of CBT outcome than genetic factors. Regardless, it would be necessary to evaluate the clinical relevance of any positive association between (epi)genetic variation and psychological treatment [21].

We also found no evidence to suggest that childhood abuse and childhood neglect predict CBT outcome. Only two previous studies have investigated the effects of childhood maltreatment on CBT outcome in anxiety disorders, specifically in SAD patients [25, 26]. Bruce *et al*. did not find an association between childhood maltreatment and CBT outcome, as Alden *et al*. had previoulsy observed. However, in the study by Bruce *et al*., the patients who reported emotional abuse, emotional neglect and sexual abuse showed greater severity before and throughout treatment. Here, we focused on symptom reduction as our index of CBT outcome, and it remains possible that other variables (e.g. attrition rates or relapse/recurrence rates) may be affected by childhood abuse/neglect. Future studies that include these and other treatment-related outcomes may provide additional insights.

Finally, we detected no interaction between the polymorphism of interest and childhood abuse or neglect. As suggested by Keers *et al*. [60], inconsistent findings in previous studies on stressful life events may be due to unmeasured genetic moderators. Again, epigenetic factors have recently been identified in individuals with a history of abuse [61] and in institutionalized children [62], and it would be interesting for future studies to add epigenetics to the prediction of psychological treatment outcomes.

Several limitations of this study should be noted. First, a limitation of reporting negative results is that they could be due to a lack of statistical power rather than a real lack of effect in the population. However, the present study reports on a larger or similar sample (N = 97) than several previous studies that have found significant effects of genetic polymorphisms (the *BDNF* Val66Met and others) on the psychological [18, 19, 63] or pharmacological treatment [64, 65, 66] of anxiety and related disorders. Our sample is also much larger than a previous study that did find an effect of chilldhood maltreatment on CBT outcome [25]. A formal estimate of power in our study was hampered by the fact that we measured the effects of treatment using response trajectories, and the previous literature could not offer sufficient guidance to provide such calculations. Be as it is, one possibility to increase the sample size in future studies would be to include patients with different anxiety-related diagnoses and/or using different CBT modalities. This strategy was followed in Lester *et al*. [58], where children with mixed anxiety disorders receiving individual CBT, group-based CBT or parent-supported guided self-help were included. Second, most of the patients were receiving pharmacological treatment during CBT. While medication was kept stable for at least two months before the treatment started, we cannot exclude the possibility that improvement in panic symptoms was due to maintaining pharmacotherapy over time. However, this was controlled for by including drug treatment as a covariate in our analyses. Third, our sample consisted mainly of non-responders to pharmacotherapy, which could represent a subgroup with different (including genetic) characteristics than the entire spectrum of patients with PD. Fourth, due to the low frequency of the *BDNF* Met allele in the European population [67], our Met/Met group was too small to be tested. Fifth, although we accounted for some environmental factors, the *BDNF* Val66Met polymorphism is unlikely to adhere to a simple genetic model [57], and so future studies involving genetic combinations may be warranted.

In conclusion, we found no evidence to support the effect of either the *BDNF* Val66Met polymorphism or childhood trauma-related variables on CBT outcome. Future studies that address the role of other (including epigenetic) variables may shed light on the neurobiological prediction of psychological treatment outcomes.

## Supporting Information

S1 Fig Mean scores of the PDSS-SR throughout the treatment and follow-up for Met-carriers and Val-Val. (JPG)
