# Correction: Predicting Response Trajectories during Cognitive-Behavioural Therapy for Panic Disorder: No Association with the BDNF Gene or Childhood Maltreatment

**DOI:** 10.1371/journal.pone.0167833

**Published:** 2016-12-01

**Authors:** Martí Santacana, Bárbara Arias, Marina Mitjans, Albert Bonillo, María Montoro, Sílvia Rosado, Roser Guillamat, Vicenç Vallès, Víctor Pérez, Carlos G. Forero, Miquel A. Fullana

The S1 Fig legend is incorrect. The MET-CARRIERS and VAL-VAL groups are swapped. The authors have provided a corrected version here.

## Supporting Information

S1 Fig Mean scores of the PDSS-SR throughout the treatment and follow-up for Met-carriers and Val-Val. (JPG)
