# Supplementary figures and images for: Predicting Response Trajectories during Cognitive-Behavioural Therapy for Panic Disorder: No Association with the BDNF Gene or Childhood Maltreatment
Source: PLoS One. 2016 Jun 29;11(6):e0158224. doi: 10.1371/journal.pone.0158224 (PMC4927091; doi:10.1371/journal.pone.0158224)

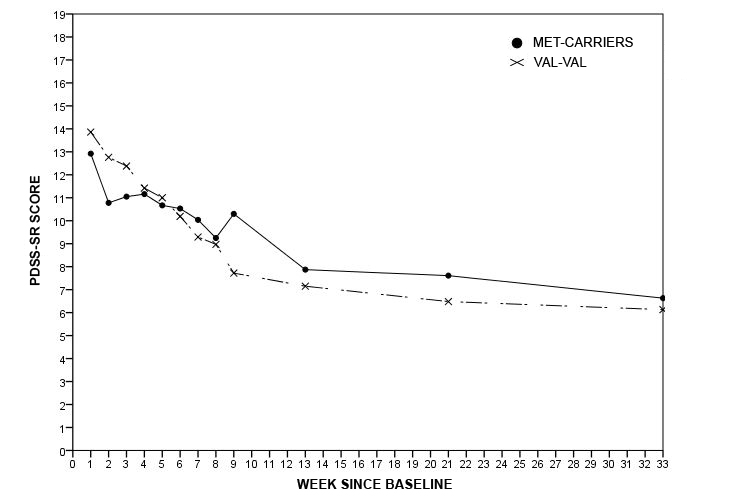

Supplement: S1 Fig — (JPG) [file pone.0158224.s001.jpg]
