# Supplementary figures and images for: Correction: Predicting Response Trajectories during Cognitive-Behavioural Therapy for Panic Disorder: No Association with the BDNF Gene or Childhood Maltreatment
Source: PLoS One. 2016 Dec 1;11(12):e0167833. doi: 10.1371/journal.pone.0167833 (PMC5132167; doi:10.1371/journal.pone.0167833)

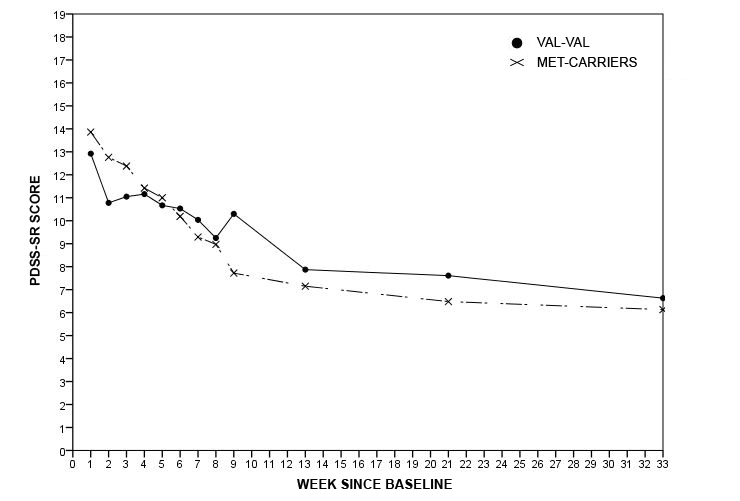

Supplement: S1 Fig — (JPG) [file pone.0167833.s001.jpg]
